# Who’s Who? Discrimination of Human Breast Cancer Cell Lines by Raman and FTIR Microspectroscopy

**DOI:** 10.3390/cancers14020452

**Published:** 2022-01-17

**Authors:** Inês P. Santos, Clara B. Martins, Luís A. E. Batista de Carvalho, Maria P. M. Marques, Ana L. M. Batista de Carvalho

**Affiliations:** 1 Molecular Physical-Chemistry R&D Unit, Department of Chemistry, University of Coimbra, 3004-535 Coimbra, Portugal; ips@uc.pt (I.P.S.); clara.b.martins@uc.pt (C.B.M.); almbc@uc.pt (A.L.M.B.d.C.); 2 Department of Life Sciences, University of Coimbra, 3000-046 Coimbra, Portugal

**Keywords:** Raman microspectroscopy, FTIR microspectroscopy, human breast cancer cells, breast cancer differentiation, triple-negative breast cancer (TNBC), TNBC breast cancer subtyping, cancer diagnosis, cancer chemotherapy

## Abstract

**Simple Summary:**

Normal-to-malignant transition in human cells is still a poorly understood process, as well as the mechanisms leading to cancer invasion and metastasis. An in-depth characterisation and understanding of the genetic and chemical variations underlying carcinogenesis and cancer progression are paramount for improving diagnosis and design better therapeutic strategies, aiming at a better prognosis for oncology patients. The present study reports the application of spectroscopic methods (micro-Raman and infrared) for identifying specific biomarkers of malignancy in several types of human breast cancer cells. The results thus obtained led to an accurate discrimination between the highly metastatic mammary tumours (triple-negative) and the less aggressive ones (non-triple negative), as well as among triple-negative subtypes.

**Abstract:**

(1) Breast cancer is presently the leading cause of death in women worldwide. This study aims at identifying molecular biomarkers of cancer in human breast cancer cells, in order to differentiate highly aggressive triple-negative from non-triple-negative cancers, as well as distinct triple-negative subtypes, which is currently an unmet clinical need paramount for an improved patient care. (2) Raman and FTIR (Fourier transform infrared) microspectroscopy state-of-the-art techniques were applied, as highly sensitive, specific and non-invasive methods for probing heterogeneous biological samples such as human cells. (3) Particular biochemical features of malignancy were unveiled based on the cells’ vibrational signature, upon principal component analysis of the data. This enabled discrimination between TNBC (triple-negative breast cancer) and non-TNBC, TNBC MSL (mesenchymal stem cell-like) and TNBC BL1 (basal-like 1) and TNBC BL1 highly metastatic and low-metastatic cell lines. This specific differentiation between distinct TNBC subtypes—mesenchymal from basal-like, and basal-like 1 with high-metastatic potential from basal-like 1 with low-metastatic potential—is a pioneer result, of potential high impact in cancer diagnosis and treatment.

## 1. Introduction

Breast cancer, a highly heterogeneous disease originating from the epithelial cells lining the milk ducts, is the second most common type of cancer and the most common in women (affecting one in every four). Despite all efforts regarding screening and treatment, this type of neoplasia, with a growing incidence in the last four decades, remains a leading cause of death among women worldwide (mainly due to bone, lung, brain and liver metastasis) [1,2]. Based on their overall morphology and structural organisation, breast tumours were classified into multiple categories, two major groups having been identified (accounting for ca. 90% of the total cases): invasive ductal and invasive lobular [3,4]. This molecular classification is based on the presence of specific markers which can define cancer subtypes, with different prognosis and distinct susceptibility to chemotherapy [5]. The most common of these biomarkers are the oestrogen receptor (ER), the progesterone receptor (PR) and the human epidermal growth factor 2 receptor (HER2) [6,7,8]. A combined assessment of the three markers (ER/PR/HER2) allows an accurate assignment to specific categories, namely ER^+^ (ER^+^/HER2^−^), HER2^+^ (ER^−^/HER2^+^), triple-negative (ER^−^/PR^−^/HER2^−^) and triple-positive (ER^+^/PR^+^/HER2^+^). Additionally, breast cancer sub-classification differentiates these tumours into five groups [9]: basal-like (BL), luminal (luminal A/HER2 negative, luminal B/HER2 negative and luminal B/HER2 positive), normal-like, HER2 and claudin-low. Particularly regarding triple-negative breast cancer (TNBC), a further discrimination was achieved into several molecular subtypes [7,10,11,12,13,14]: basal-like 1 (BL1), basal-like 2 (BL2), mesenchymal (M), mesenchymal stem cell-like (MSL), immunomodulatory (IM), luminal androgen receptor (LAR) and an unstable subtype (UNS).

The ER^+^ breast tumours are those with a best overall outcome, while TNBC (the majority of which are basal-like) are the ones with the poorest prognosis [15,16], along with inflammatory breast cancer (a rare and very aggressive type of tumour). TNBC accounts for ca. 15% of all breast cancers, being more prevalent in premenopausal young women (under 40 years old) [17,18]. Due to its aggressiveness and metastatic potential (despite surgical removal of the primary tumour), it has a high risk of recurrence leading to mortality in the first 5 years after diagnosis [17,19,20]. The overall survival for women with TNBC is 13.3 months, and less than 30% of the cases survive longer than 5 years [15,18]. While the average time relapse for non-TNBC is 35–67 months, in TNBC patients it is only 19–40 months [18]. Accordingly, the development of novel chemotherapeutic strategies against triple-negative breast cancer is an urgent clinical need, specifically aiming at a higher efficacy regarding cell growth inhibition and decreased angiogenesis and invasiveness, coupled to lower acquired resistance and deleterious side effects.

An early diagnosis is critical for identifying the exact type/subtype of neoplasm with a view to choose the most effective treatment approach, thus improving chemotherapy success and survival rates of oncology patients. This relies on a thorough knowledge of the molecular features of the different types of cancer, that will allow an accurate discrimination in a rapid and reliable way. The transition from normal to malignant state and between non-invasive and invasive tumours, are still poorly understood processes. This type of cell transformation is associated with chemical and morphological variations. Particularly regarding breast cancer (mainly TNBC), current data suggests that its initiation and progression may be associated to epigenetic alterations (such as DNA methylation or chromatin remodelling) [17,21]. Recent studies have focused on the biophysics of the cancer state, shedding a new light on carcinogenesis beyond the recognised biochemical and genetic variations associated to malignancy. A strong correlation between neoplasia and cellular plasticity has been identified, cancer cells displaying an enhanced deformability relative to non-malignant ones [22,23,24,25] which seems to favour uncontrollable growth. Changes in the dynamical activity of intracellular water between healthy and cancerous human cells were recently probed by the authors (using neutron scattering techniques) [25]. However, it is still not clear how these chemical and dynamical changes are produced and how they accumulate in cells, particularly within the tumour microenvironment.

Raman and Fourier transform infrared (FTIR) vibrational spectroscopies are promising tools in medicinal chemistry, since they are non-invasive, reproducible, cost-effective and highly accurate analytical techniques that enable rapid measurements and deliver unique molecular level signatures of biospecimens with high accuracy, sensitivity and specificity. Instead of monitoring morphological differences as in current histopathological methods, they probe chemical variations which usually arise earlier. Coupled to optical microscopy, they allow to interrogate heterogeneous biological samples with unmatched sub-cellular spatial resolution. They are label-free, deliver chemical image maps apart from average biochemical information and can be reliably applied to both in vitro and in vivo conditions. Hence, there is an increasing number of reported studies on the application of infrared and Raman spectroscopies to monitor cells, both live and fixed (e.g., healthy from cancer discrimination and metabolic response to drugs) and tissues (e.g., cancer diagnosis and treatment monitoring) [4,26,27,28,29,30,31,32,33,34,35,36,37,38,39,40,41,42,43,44]. Thanks to their distinctive characteristics and ease of automation, these techniques constitute an improved approach for cancer detection when compared to the currently used diagnostic methods such as the gold standard histopathological assessment (which lacks sensitivity and specificity at an early stage of the disease). Therefore, their application in the clinical arena may greatly accelerate point-of-care decisions and improve patient outcomes.

The aim of the present study was to take a step forward and apply complementary Raman and FTIR microspectroscopy (upon multivariate analysis) to unveil specific and sensitive molecular biomarkers of cancer in human neoplastic breast cells, assessing whether these techniques are able to detect even more subtle differences than those between malignant and non-malignant cells. Accordingly, an unambiguous discrimination was sought between: (i) triple-negative and non-triple-negative; (ii) different TNBC subtypes, namely mesenchymal vs. basal-like and (iii) TNBC basal-like 1 with high-metastatic potential vs. TNBC basal-like 1 with low-metastatic potential. The results thus obtained should provide particular features of malignancy and identify specific biochemical reporters, which will contribute to differentiate different types of human breast carcinoma. This constitutes a paramount advance in cancer diagnosis, particularly regarding a type of malignancy with high morbidity and mortality.

## 2. Materials and Methods

The list of chemicals as well as the details of the cell culture procedure, Raman and FTIR microspectroscopy measurements, spectral pre-processing and multivariate analysis are described in the Supplementary Materials.

### 2.1. Cell Lines

Four human breast cancer cells were investigated in this work: (i) triple negative (TNBC)-MDA-MB-231 ductal carcinoma, claudin-low/MSL, metastatic; MDA-MB-468 ductal carcinoma, BL1, high-metastatic potential; and HCC-1143 invasive ductal carcinoma, BL1, low-metastatic potential; (ii) non-triple-negative—MCF-7 adenocarcinoma, luminal A/HER2 negative, metastatic.

### 2.2. Sample Preparation for Spectroscopic Analysis

Upon harvesting by trypsinisation, the cells were centrifuged and the pellet was resuspended in culture medium and seeded at a concentration of 3 × 10^4^ cells cm^−2^ onto optical substrates suitable for either Raman [45] or FTIR data collection [46]: respectively MgF_2_ (2 × 20 mm) or CaF_2_ (UV-grade, 1 × 13 mm) disks, previously cleaned with 70% ethanol and sterilised.

After a 24 h incubation period (allowing the cells to attach), the growth medium was removed and the cells were washed twice with phosphate buffered saline (PBS), upon which they were fixed in 4% formalin (diluted in NaCl-0.9% from the commercial 10% neutral-buffered formalin solution) for 10 min [46]. After repeated washing with pure water (to remove any residual salt), the disks were allowed to air-dry (at room temperature) prior to spectroscopic analysis.

All samples were prepared in triplicate, in a single experiment.

### 2.3. Raman Microspectroscopy

The Raman spectra were recorded in a WITec confocal Raman microscope system alpha 300R (WITec GmbH, Ulm, Germany) with an automated sample stage, coupled to an ultrahigh-throughput spectrometer UHTS 300Vis-NIR, using a frequency-doubled Nd:YAG exciting laser line, at 532 nm.

### 2.4. FTIR Microspectroscopy

The microFTIR spectra were acquired in the mid-IR interval (400–4000 cm^−1^), in transmission mode, using a Bruker Optics Vertex 70 FTIR spectrometer coupled to a Bruker Hyperion 2000 microscope, both purged by CO_2_-free dry air.

Both FTIR and Raman data were captured as single point spectra, each spectrum recorded on a different single cell, across a cell population comprising randomly distributed cells displaying a high heterogeneity.

## 3. Results

### 3.1. All Breast Cancer Cells—Overall Characterisation

Table 1 comprises the Raman and FTIR experimental wavenumbers obtained for the cancer cells under study, reflecting their biochemical profile (mainly signals from DNA, proteins, lipids and polysaccharides) and high heterogeneity. The FTIR and Raman fingerprint spectra of each cell line were pre-processed and normalized (Figure S1).

**Figure 1:**
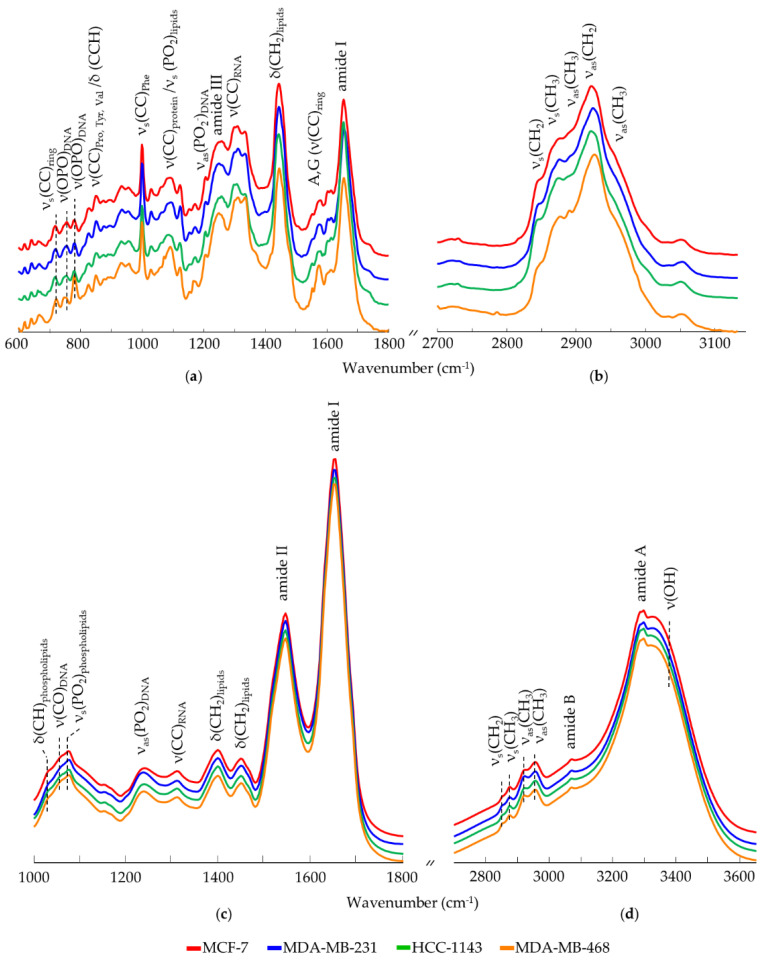
Mean Raman spectra (600–1800 cm^−1^ and 2700–3140 cm^−1^, (**a**,**b**) and mean FTIR spectra (1000–1800 cm^−1^ and 2700–3650 cm^−1^, (**c**,**d**)) for the human breast cancer cell lines MDA-MB-231, MDA-MB-468, HCC-1143 and MCF-7. (δ—deformation; ν—stretching; s—symmetric; as—anti-symmetric. Phe—phenylalanine; Pro—proline; Tyr—tyrosine; Val—valine. For clarity’s sake, the mean spectra are offset along the y-axes).

Mean spectral biochemical signatures were generated for each type of cell line, by averaging the corresponding Raman or FTIR spectra acquired from different cellular locations (Figure 1a–d, respectively).

The cells were chemically fixed with a 4% formaldehyde solution (formalin). Formalin causes crosslinking between the aldehyde and the primary and secondary amine groups of cellular proteins, thus retaining the cellular constituents in a close to in vivo relationship. It is expected that formalin fixation procedure induces some reduction of vibrational band intensities, related to the disruption of lipid assembly and conformational protein changes. Nevertheless, the fixation has shown to have a weak impact on the overall molecular content and yielding the spectral profile closest to that observed in the live state. This being a comparative study, all cell lines were virtually equally affected by the fixation process, which does not impact on the discrimination attained between the distinct types of cancer cell lines under the same conditions.

The spectral regions that showed statistically significant differences across cell lines comprise contributions from the major cellular components (Table 1): nucleic acids, lipids (including membrane phospholipids), proteins and carbohydrates (876, 934, 1030, 1062 cm^−1^). Significant variations were detected in the Raman profiles, namely regarding the bands assigned to the modes ν(CC)_DNA/bases_ (*ca.* 780 cm^−1^), ν(CC)_RNA_ (*ca.* 1302 cm^−1^), ν(C=C)_proteins/lipids_ (*ca.* 1580 cm^−1^) and ν(CH_2_)_proteins/lipids_ (*ca.* 2850 cm^−1^), the main differences having been observed for the TNBC BL1 ductal carcinoma (MDA-MB-468) relative to the other cell lines under analysis. Protein (Amide I and III, respectively at 1650 and 1272 cm^−1^) and lipid ratios were found to vary among these breast cancer subtypes. Specifically, regarding the protein spectral profile, these variations are ascribed to the different structures and conformations present within the cell (mainly α-helix or β-sheet) [47,48]. In addition, proteins are regarded as important indicators in cancer biology, since protein metabolism is closely associated to cell proliferation and division, transcription (RNA synthesis), translation (protein synthesis) and migration processes [4,48]. Some authors have reported a predominance of proteins in abnormal tissue, both benign and malignant [28,48,49]. In addition, Mohamed and co-workers [35] verified good discrimination between two TNBC cell lines (MDA-MB-231 and SUM-149) mainly based on the spectral signature of proteins, nucleic acids and amino acids. Furthermore, the characteristic CH_2_/CH_3_ stretching modes (namely from lipid carbon chains), detected at high wavenumbers (Table 1), are considered relevant spectral biomarkers for the identification of distinct types of cancer [48]. These modes are known to vary considerably regarding the type (e.g., unsaturation degree) and amount of cellular lipids (e.g., membrane fatty acids). Studies by Ozek and co-workers [50] highlighted the influence of high amounts of polyunsaturated fatty acids on the transport and signalling pathways, associated to cell reshaping and adhesion processes that determine metastatic ability. The current study revealed an increased amount of lipids in the two TNBC BL1 cell lines (MDA-MB-468 and HCC-1143), which is in accordance with the infrared and Raman data formerly obtained by the authors for the TNBC MSL MDA-MB-231 cell line [33] that evidenced a high lipid content, particularly regarding glycerophospholipids known to be augmented in cancer cells [51]. In addition, other studies have reported an increase in the lipid content in breast cancer cells as compared to normal breast cells [43,52]. Several authors have shown a correlation between the rise in cytoplasmic lipid droplets and the increase in cancer aggressiveness [38,52,53]—the number of lipid droplets in non-malignant breast cells being reported as two- and four times lower than in non-TNBC and TNBC cells, respectively. In addition, a recent Raman study of human breast cancer cell lines (MDA-MB-231, MDA-MB-435s and SK-BR-3) [38] also detected a noticeable lipid expression in the MDA-MB-231 TNBC cells when compared to the non-TNBC (luminal HER2^+^) SK-BR-3, which was suggested to be due to an increased content in unsaturated fatty acids (mostly within the membrane). It should be emphasised, however, that the epithelial MDA-MB-435s cell line was wrongly included in this study since it was found to have undergone phenotypic and genotypic drift thus expressing melanoma-specific genes [54,55,56,57].

**Figure 2:**
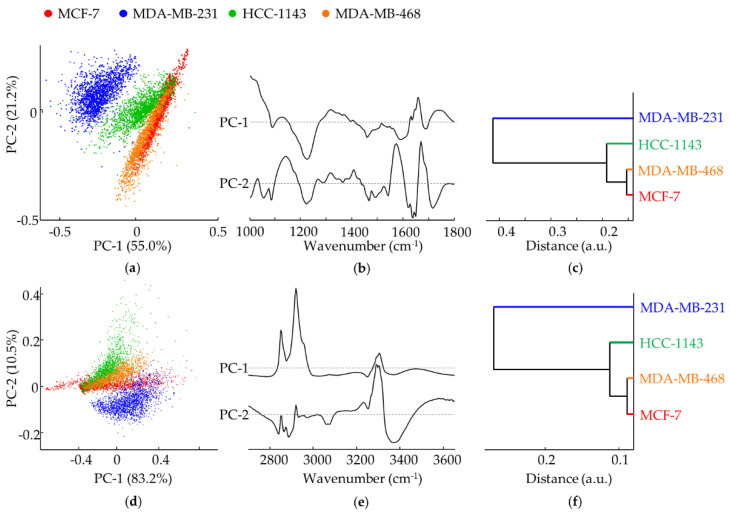
PCA of FTIR data for all cell lines studied (MCF-7, MDA-MB-231, HCC-1143 and MDA-MB-468). (**a**,**b**) Scores and loading plots of the fingerprint region (1000–1800 cm^−1^). (**c**) Hierarchical clustering-derived dendrogram of the mean fingerprint FTIR spectra for the four cell lines. (**d**,**e**) Scores and loading plots of the high wavenumber region (2700–3650 cm^−1^). (**f**) Hierarchical clustering-derived dendrogram of the mean high-wavenumber FTIR spectra for the four cell lines. For clarity’s sake the spectral loadings are offset, the dashed horizontal lines indicating zero loading.

**Figure 3:**
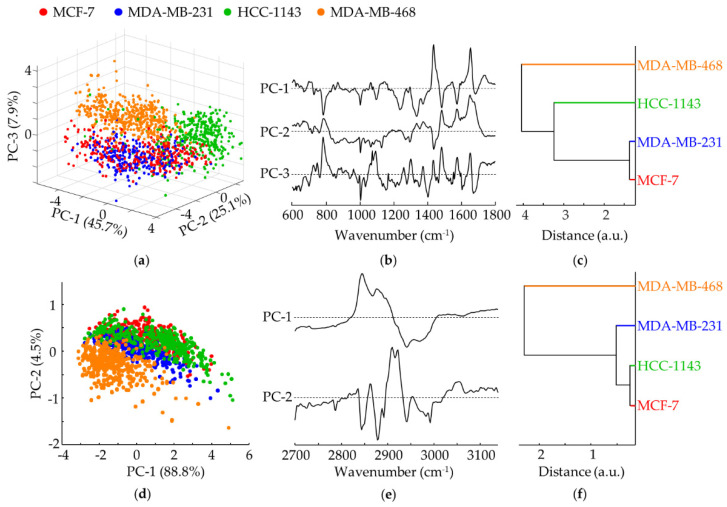
PCA of Raman data for all cell lines studied (MCF-7, MDA-MB-231, HCC-1143 and MDA-MB-468). (**a**,**b**) Scores and loading plots of the fingerprint region (600–1800 cm^−1^). (**c**) Hierarchical clustering-derived dendrogram of the mean fingerprint Raman spectra for the four cell lines. (**d**,**e**) Scores and loading plots of the high wavenumber region (2700–3150 cm^−1^). (**f**) Hierarchical clustering-derived dendrogram of the mean high-wavenumber Raman spectra for the four cell lines.

The chemical differences among cell lines were clearly unveiled upon PCA analysis of the spectroscopic results—both FTIR and Raman (Figure 2 and Figure 3, respectively). Overall, the FTIR data allowed to differentiate the TNBC cell lines from the non-TNBC (MDA-MB-231, MDA-MB-468 and HCC-1143 vs. MCF-7) (Figure 2a–f). In addition, there was a very good discrimination between TNBC mesenchymal and basal-like cells (MDA-MB-231 vs. HCC-1143). Major differences in the spectral fingerprint region were along PC-1, explaining 55.0% of the total data variance. The loading plots corresponding to each principal component provided information on the main biochemical differences between cells, mainly along PC-1: a higher contribution from ν_as_(PO_2_^−^)_DNA_, α(CH_2_)_lipids_ and ν(C=O)_aminoacid/side chain_ (at 1238, 1450 and 1690 cm^−1^, respectively) for MDA-MB-231 (TNBC MSL); a lower input from Amide I/ν(C=O)_peptid bond_ and ν(C=O)_ester/phospholipids_ (at 1650 and 1730–42 cm^−1^, respectively) for MDA-MB-231 and HCC-1143 (TNBC MSL and TNBC BL1—low-metastatic potential) relative to MDA-MB-468 and MCF-7 (TNBC BL1—high-metastatic potential and non-TNBC). Analysis along PC-2 did not yield a significant distinction, MCF-7 and MDA-MB-468 showing a wider dispersion relative to the TNBC cell lines MDA-MB-231 and HCC-1143: the bands ascribed to ν(CC), ν(CN), ν(CO) and ρ(CH_2_) from proteins and carbohydrates, and ν(=C-C=)_conjugated_ from lipids (at 1157 cm^−1^) displayed the strongest contribution for the TNBC MSL cell line (MDA-MB-231) and the weakest for TNBC BL1 MDA-MB-468, as opposed to ν(C=O)_B-DNA_ (at 1717 cm^−1^) which showed an inverse trend; in turn, α(CH_2_)_membrane lipids_ (at 1450 cm^−1^) contributed more for the HCC-1143 cells (TNBC BL1—low-metastatic potential) and less for the MDA-MB-468 (TNBC BL1—high-metastatic potential). Regarding the high-wavenumber range the major differences between cell-lines were detected along PC-2 (mainly for MDA-MB-231), explaining 10.5% of the total data variance: the ν(CH_2_)_lipids, proteins_ vibrational modes (at ca. 2850, 2919 cm^−1^) appear to be the main responsible for the separation of the TNBC MSL cell line (MDA-MB-231) from all the others; a stronger contribution from ν_s_(CH_3_) (at 2877 cm^−1^) and a lower one from ν(NH)_amide A_ (at 3250–90 cm^−1^) was detected for MDA-MB-231; in addition, the Amide A signal was predominant for the TNBC BL1 cells (MDA-MB-468 and HCC-1143).

From the dendrograms obtained from the average FTIR spectra (Figure 2c,f) a clear differentiation is observed between MDA-MB-231 and the other cell lines, MCF-7 and MDA-MB-468 showing the highest similarity (in both spectral regions). It is also evident that the cluster distance scale shown in the first dendrogram is larger, evidencing that the underlying variability of the fingerprint region is more relevant for this discrimination.

The Raman data (Figure 3a–f) yielded a much less significant differentiation between cell lines when compared to infrared. In the fingerprint interval the major differences were found along PC-1 followed by PC-3 (explaining 45.7% and 7.9% of the total data variance, respectively), while in the high wavenumber region discrimination was mainly along PC-2 (4.5% of the total data variance). Separation was evident only for the TNBC BL1 cells (MDA-MB-468 and HCC-1143), based on the signals from ν(CC)_DNA/bases_ (at ca. 780–800 cm^−1^), ν(PO_2_^−^)_DNA_ (at 1092 cm^−1^), α(CH_2_)_lipids_ (at ca. 1450 cm^−1^), Amide I (at 1650 cm^−1^) and ν_s_(CH_2_/CH_3_) (at 2846–2964 cm^−1^).

The dendrograms obtained from the average Raman spectra (Figure 3c,f) reveal that MDA-MB-468 differentiates well from the other cell lines. Considering the fingerprint region-derived dendrogram, MCF-7 and MDA-MB-231 are the cell lines that show the highest similarity, as opposed to the high-wavenumber region where the cell lines with the highest resemblance are HCC-1143 and MCF-7. Again, the distance scale shown in the dendrogram from the fingerprint region is larger, reflecting the highest variability in this spectral region.

Table 2 and Table 3 represent the confusion tables of the RF classification model validation for the FTIR and Raman data, respectively. The rows correspond to the cell lines true class and the columns correspond to the results of the classification model. The values along the primary diagonal represent the number of correctly classified spectra. Regarding the FTIR classification model (Table 2), sensitivities of 68.6%-99.7%, specificities of 90.9%-100% and overall accuracies of 91.4–99.9% were obtained. MDA-MB-468 is the cell line with the highest number of misclassifications while MDA-MB-231 is the one with less misclassified spectra. The highest number of incorrect classifications occurred between MCF-7 and MDA-MB-468, followed by MCF-7 and HCC-1143 and by MCF-7 and MDA-MB-468. This is expected given the PCA scores plot shown in Figure 2a, where MCF-7 and MDA-MB-468 show the lowest separation, and the hierarchical clustering-derived dendrogram showing the shortest cluster distance (Figure 2c).

For the Raman classification model, sensitivities of 63.6%–94.3%, specificities of 89.8%–97.2% and overall accuracies of 85.8–95.8% were obtained (Table 3), the latter being lower than those obtained with FTIR. MDA-MB-231 is the cell line with the highest number of misclassifications (with a limited sensitivity of 63.6% and specificity of 93.8%), and HCC-1143 and MDA-MB-468 present the lowest number of misclassified spectra (with sensitivities and specificities above 92% and an overall accuracy above 95%). The most incorrect classifications were found for MDA-MB-231, which was misclassified as MCF-7. This result was expected given the PCA scores plot shown in Figure 3a (MCF-7 and MDA-MB-231 not discriminated along either PC-1, PC-2 or PC-3). Although the number of spectra per class did not vary substantially in this study, and a large number of spectra were used, a careful analysis must be performed when using decision trees algorithms given their sensitivity to class imbalance.

### 3.2. TNBC versus Non-TNBC Cells

**Figure 4:**
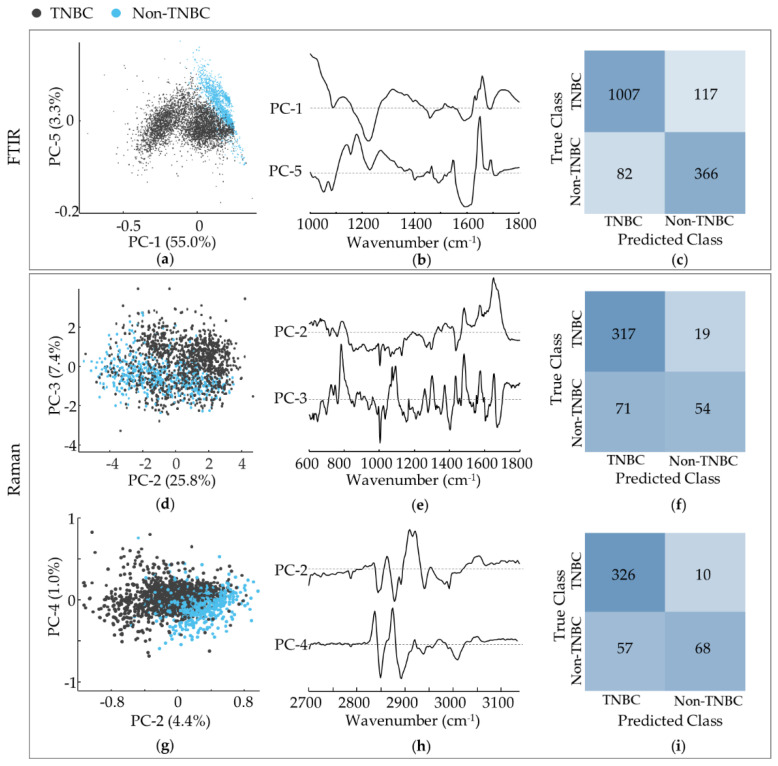
PCA of FTIR and Raman data for TNBC vs. non-TNBC cells. (**a**,**b**) Score and loading plots of the FTIR fingerprint region. (**c**) Confusion table of the FTIR classification model tested on independent data. (**d**,**e**) Scores and loading plots of the Raman fingerprint region. (**g**,**h**) Scores and loading plots of the Raman high wavenumber region. (**f**,**i**)—Confusion tables of the Raman fingerprint (**f**) and high wavenumber (**i**) classification models tested on independent data.

A PCA analysis was performed for the vibrational data obtained for TNBC (MDA-MB-231, HCC-1143 and MDA-MB-468) as compared to non-TNBC (MCF-7) cell lines (Figure 4a–i), enabling to discriminate between them. For the infrared fingerprint region, separation was obtained along PC-1 (upon a PC-1 vs. PC-5 combination, representing 55.0% and 3.3% of the total data variance, respectively) based on: a higher contribution from ν_as_(PO_2_)_DNA_ (at 1238 cm^−1^) coupled to a lower input from ν(CC)_RNA/bases_ (at 1302 cm^−1^) and Amide I (at 1650 cm^−1^) for TNBC cells. Regarding the Raman data, PC-2 explains 25.8% of the total data variance for the fingerprint interval, followed by PC-3 (7.4%), the separation between triple-negative and non-triple negative cells being more evident along PC-3, mainly according to: ν(CC)_DNA/bases_ (at ca. 780–800 cm^−1^), Amide I (at ca. 1650 cm^−1^) and ν(PO_2_^−^)_DNA_ (at 1092 cm^−1^), which contribute more significantly for the TNBC cells, while α(CH_2_) (at 1450 cm^−1^) is predominant for non-TNBC. For the Raman high wavenumber range, in turn, separation between triple-negative and non-triple-negative cell lines occurs along PC-2 (4.4%) based on the ν_s_(CH_2_/CH_3_) modes (at ca. 2846–2970 cm^−1^), possibly due mostly to variations in the lipidic cellular content (which are known to be closely linked to invasiveness and metastatic ability, that characterise TNBC).

Figure 4c,f,i shows the confusion tables of the FTIR classification models concerning the comparison between TBNC and non-TNBC cell lines. Spectra from TNBC achieved a higher percentage of correct classification than spectra from non-TNBC. Considering TNBC as the positive class, a sensitivity of 89.6% and a specificity of 81.7 % were obtained, with an overall accuracy of 87.3%. The Raman results led to higher sensitivities (94.4% and 97.0% for the fingerprint and high wavenumber regions, respectively), at the cost of poorer specificities (43.0% and 54.4% for the fingerprint and high wavenumber regions, respectively), with an overall accuracy of 80.5% and 85.4% respectively for the fingerprint and high wavenumber regions). This reflects the incomplete TBNC vs. non-TNBC separation already evidenced in the corresponding PCA scores (Figure 4d,g).

### 3.3. TNBC—Mesenchymal Stem-Cell Like versus Basal-like 1 Cells

**Figure 5:**
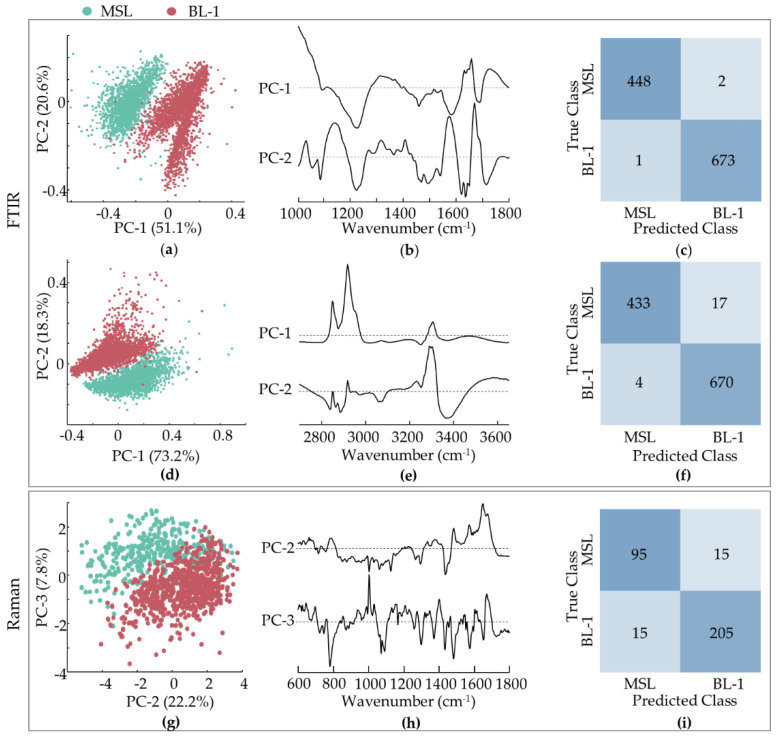
PCA of FTIR and Raman data for TNBC MSL vs. TNBC BL1 cell lines. (**a**,**b**) Score and loading plots of the FTIR fingerprint region. (**d**,**e**) Scores and loading plots of the FTIR high wavenumber region. (**c**,**f**) Confusion tables of the fingerprint (**c**) and high wavenumber (**f**) FTIR classification models tested on independent data. (**g**,**h**) Scores and loading plots of the Raman fingerprint region. (**i**) Confusion table of the Raman classification model tested on independent data.

The results gathered for the mesenchymal stem cell-like (MDA-MB-231) and basal-like (MDA-MB-468 and HCC-1143) triple-negative breast cancer cell lines were analysed by PCA and compared (Figure 5a–i). A clear separation was achieved between these two groups by FTIR and Raman. For the former, discrimination was observed along PC-1 for the fingerprint interval (51.1%) and along PC-2 for the high wavenumber range (18.3%), based on the following spectral differences reflected in the loading plots (Figure 5b,e): a higher contribution from ρ(CH_2_)_phospholipids_ (at 1030 cm^−1^), Amide I (at ca. 1650 cm^−1^), ν(C=O)_ester/phospholipids_ (at 1730–42 cm^−1^) and Amide A (ν(NH)_peptidic_ (at 3250–90 cm^−1^), combined with a smaller influence from ν(=CC=)_conjugated/lipids_ (at 1157 cm^−1^) and (CH_2_)_lipids_ (at 1450 and 2846–78 cm^−1^) for BL1 cells as compared to MSL. Concerning the Raman data, a MSL from BL1 separation was attained by combining PC-2 and PC-3, based on: ν(CC)_DNA/bases_ (at ca. 720 and 754 cm^−1^), ν(CO)_DNA/deoxyribose_/ν(CC/CN)_proteins/lipids_ (at 1062 cm^−1^) and α(CH_2_)_lipids_ (at 1450 cm^−1^) more significant for MSL, as compared to ν(OPO)_DNA/backbone_ (at 780 cm^−1^) and Amide I (at 1650 cm^−1^), higher for BL1.

This discrimination between mesenchymal- and basal-like TNBC subtypes is noteworthy and suggests a significant biochemical variation among them. To the best of the authors´ knowledge, this is the first report of such a differentiation based on FTIR and Raman microspectroscopic techniques.

Figure 5c,f,i depicts the confusion tables of the classification models concerning the MSL vs. TBNC BL1 cells. Regarding the FTIR data, sensitivities of 99.6% and 96.2%, and specificities of 99.9% and 99.4%, were achieved for the fingerprint and high-wavenumber regions, respectively (considering MSL as the positive class). The number of misclassified spectra were lower in the fingerprint region than in the high wavenumber interval (only 3 misclassified spectra in a total of 1124). The overall accuracy was 99.8% and 98.1%, for the fingerprint and high wavenumber ranges, respectively. The Raman results were reflected a slightly less clear separation (Figure 5g), with a sensitivity of 86.4%, a specificity of 93.2%, and an overall accuracy of 90.9%. A higher percentage of Raman spectra from MSL cells were misclassified when compared to TNBC BL1.

### 3.4. TNBC Basal-like 1 Cells—High-Metastatic versus Low-Metastatic Potential

**Figure 6:**
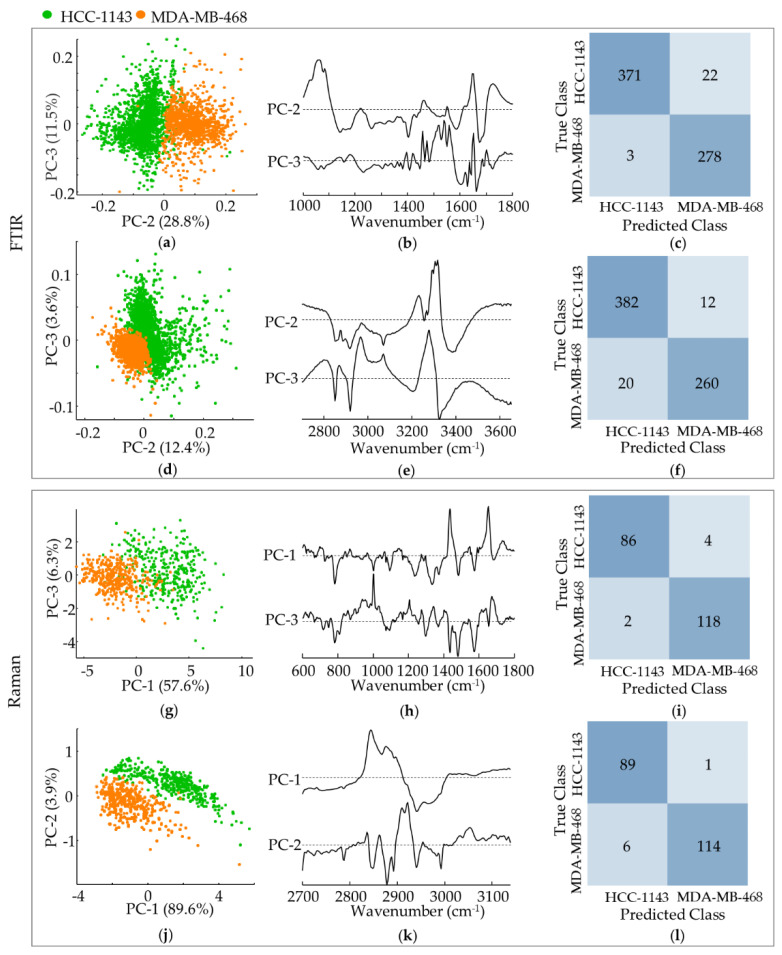
PCA of FTIR and Raman data for TNBC BL1/high-metastatic potential (MDA-MB-468) vs. TNBC BL1/low-metastatic potential (HCC-1143) cell lines. (**a**,**b**) Scores and loading plots of the FTIR fingerprint region. (**d**,**e**) Scores and loading plots of FTIR high wavenumber region. (**c**,**f**) Confusion tables of the fingerprint (**c**) and high wavenumber (**f**) FTIR classification models tested on independent data. (**g**,**h**) Scores and loading plots of the Raman fingerprint region. (**j**,**k**) Scores and loading plots of the Raman high-wavenumber region. (**i**,**l**) Confusion tables of the fingerprint (**i**) and high wavenumber (**l**) Raman classification models tested on independent data.

Apart from being able to differentiate MSL from BL1 triple-negative breast cancer cells, discrimination between the TNBC BL1 cell lines under study was also attained—high-metastatic potential MDA-MB-468 vs. low-metastatic potential HCC-1143 ductal carcinomas (Figure 6a–l).

According to the infrared results this separation was evident along PC-2 (explaining 28.8% and 12.4% of the data variance respectively in the fingerprint and high wavenumber regions), mainly based on: a higher contribution from ν(CC/CO)_DNA/lipids/carbohydrates_/ν(CN)_proteins_/δ(OCH)_carbohydrates_ (at 1062 cm^−1^), Amide I (at ca. 1650 cm^−1^) and ν(CH_2_)_lipids/proteins_. (at ca. 2850 and 2919 cm^−1^) for metastatic MDA-MB-468, coupled to a smaller input from t(NH_2_)_proteins_ (at 1400 cm^−1^) and Amide A (at ca. 3290 cm^−1^). Concerning the Raman data, MDA-MB-468 from HCC-1143 discrimination occurred along PC-1 for the fingerprint interval (57.6%) and along PC-2 for the high wavenumber range (3.9%), centred on: a larger influence from ν(CC)_DNA bases_ (at 780, 1334 and 1372 cm^−1^), Amide III_β sheet_ (at 1234 cm^−1^), ω(CH_2_)_carbohydrates_ δ_s_(CH_3_)_glycoproteins/acyl chains_ (at 1372 cm^−1^) and ν_s_(CH_2_)_lipids/proteins/carbohydrates_ (at ca. 2848 cm^−1^) for MDA-MB-468, combined to a lower contribution from α(CH_2_)_lipids_ (at ca. 1440 cm^−1^) and ν_as_(CH_2_)_lipids/proteins/carbohydrates_ (at 2924–30 cm^−1^).

Figure 5c,f,i depicts the confusion tables of the classification models for the HCC-1143 vs. MDA-MB-468 cells. Regarding the FTIR data the classification accuracy was high, with sensitivities of 94.4% and 97.0%, and specificities of 98.9% and 92.9%, respectively for the fingerprint and high-wavenumber regions (considering HCC-1143 as the positive class). For Raman, the percentage of correctly classified spectra was even higher (both in the fingerprint and high-wavenumber intervals), reflecting the clearer separation shown in Figure 6g,j. Sensitivities of 95.6% and 98.9%, and specificities of 98.3% and 95.0% were obtained (respectively for the fingerprint and high wavenumber regions).

## 4. Discussion

Cancer cells display a high intertumour heterogeneity—between different types of tumour, distinct stages of neoplastic progression or even across geographical regions—not yet fully understood at the molecular level. This heterogeneity is due to both intrinsic and extrinsic parameters, such as genomic instability, epigenetic alterations, plastic signal transduction, stemness (ability for self-renewal and differentiation) and/or variations within the tumour microenvironment (e.g., hypoxia, vascularisation and inter-cellular interactions). The phenotypic diversity of cancer cells is correlated to their migratory and metastatic nature, as well as to chemoresistance [52,58,59], and has severe implications on patients´ prognosis since metastasis is one of the main reasons of cancer-related mortality and drug resistance is a major limiting factor in therapy response and clinical outcome. Furthermore, the multiple morphological signatures of cancer cells, marked by distinct morphodynamics, have been found to be associated to their ability to invade (upon epithelial-to-mesenchymal transition (EMT)) and migrate to distant *loci* [52,58]. A molecular level approach on this intra-tumour heterogeneity, such as the one currently performed, will allow to determine the main chemical differences between distinct types (and sub-types) of cancer, and is expected to help understand tumour evolution and assist in diagnosis and treatment.

Breast cancer, in particular, is a highly heterogeneous disease triggered by a variety of genetic alterations in mammary epithelial cells. It is associated to a large cell plasticity and a remarkable ability to adapt to changing physiological conditions (through rapid genetic/epigenetic reprogramming and transcriptomic/proteomic changes) [8,60]. Among breast tumours, TNBC´s are those with a largest heterogeneity and highest mortality rates. To this date, there are no reliable biomarkers to identify these types of tumours, nor FDA-approved targeted therapies available for TNBC patients. The differentiation presently obtained between TNBC and non-TNBC cell lines is therefore a relevant result with a potential high clinical impact. This distinction was mainly based on DNA and protein vibrational signatures, which were found to predominate in the former while the signals from lipids appeared to be prevalent in non-TNBC cells. This agrees with the enhanced cell cycle and cell division constituents and pathways typical of triple-negative cancer cells.

Discrimination within the TNBC MSL and BL1 cell lines, in turn, rested on major DNA and lipid contributions for the mesenchymal stem cell-like MDA-MB-231 cells, as compared to a higher influence of proteins and membrane phospholipids for the basal-like MDA-MB-468 and HCC-1143 cells. These findings are compatible with the increased expression of genes involved in cell proliferation and differentiation, cell motility and growth signalling pathways in TNBC MSL tumours. In fact, the MDA-MB-231 cells, displaying a stellate morphology, are the most aggressive TNBC cells based on a low expression of cell-cell adhesion genes (low claudin levels, a key protein component of cellular junctions) coupled to a very high expression of epithelial-to-mesenchymal transition genes [14,61,62].

Finally, separation between the TNBC basal-like low and high-metastatic potential cell lines was established upon the larger impact of the protein constituents in the metastatic MDA-MB-468 ductal carcinoma (displaying a grape-like morphology) relative to HCC-1143. Actually, matrix enzymes such as metalloproteinases (MMPs) and heparanase are responsible for degradation of the extracellular matrix, which prompts epithelial-to-mesenchymal transition thus facilitating cancer cell motility, invasion and metastasis characteristic of the MDA-MB-468 cells.

## 5. Conclusions

Single-point FTIR and Raman acquisition were performed on different human breast cancer cells, delivering specific vibrational spectroscopic signatures that allowed us to identify biochemical changes between cellular subtypes with high accuracy.

Upon a chemometric (PCA) analysis, several degrees of discrimination were achieved among the cell lines under study: TNBC from non-TNBC, TNBC MSL from TNBC BL1 and TNBC BL1 with either low-metastatic or high-metastatic potential. Actually, besides a reliable detection of triple-negative human breast cancer, a highly specific differentiation between distinct subtypes of TNBC was obtained, which is an innovative and highly promising result, particularly aiming at an application in the clinics. The results currently obtained give an important insight over the expected subtle spectral changes among TNBC cell lines, bringing complementary knowledge to the already documented differences between normal and malignant tissues.

This molecular approach to a highly heterogeneous tumour such as breast cancer may greatly contribute to gain an in-depth understanding of the molecular changes underlying cell plasticity during neoplastic progression, which will hopefully allow an early identification of low prognosis breast cancers and the development of novel therapeutic strategies leading to an improved clinical outcome.

These results may also pave the way for a bench-top to bedside translation of FTIR and Raman microspectroscopy techniques, in a near future (to assist histopathological analysis), within the current efforts to apply cutting-edge technologies in personalised health oncology.

## Figures and Tables

**Table 1:** Raman and FTIR experimental data for the human breast cancer cell lines MDA-MB-231, MDA-MB-468, HCC-1143 and MCF-7.

Band (cm^−1^)	Assignment *^a^*			
	Nucleic Acids	Proteins	Lipids	Carbohydrates
640–642		ν(CS), τ(CC)_Tyr_		
670	(ν(CC)_ring_)_B-DNA/G, T_			
698	(ν(CC)_ring_)_B-DNA/dG_	ν(CS)_Met_		
718–722	(ν_s_(CC)_ring_)_B-DNA/A_	(ν_s_(CC)_ring_)_Trp_		
754–756	(ν(CC)_ring_)_B-DNA/dT_ ν(OPO)_DNA_	(ν_s_(CC)_ring_)_Trp_		
780–800	(ν(CC)_ring_)_C, T, U_			
826–830	(ν(OPO)_backbone_)_B-DNA_	ν(CC)_Pro, Tyr_		
850–852		ν(CC)_Pro, Tyr, Val_, δ(CCH)		ϒ(COC)_polysaccharides_
874–878	(ribose, ν(CC)_ring_)_RNA_	(ν(CC)_ring_)_Tyr_, (ν(CC))_Hyp_	ν_s_(CCN^+^)_phosphocholine_	ν(CC), ν(C-O)_ring_
890–898		ρ(CH_2_)		
934–936		ν(CC)_(α-helix)_	ν(COC)_glycolipids_	ν(COC)_glycosidic_
1000		(ν_s_(CC)_ring_)_Phe_		
1030		δ(CH)_Phe_, ν(O-CH_3_)	ν(CC), ρ(CH_2_)_phospholipids_	ν(CC), ν(CO), ν(C-OH)
1062	ν(CO)_B-DNA/deoxyribose_	ν(CC), ν(CN)	ν(CC), ν(CO)	ν(CC), ν(CO), δ(OCH)
1076–1079		ν(CC), ν(CN)	ν_s_(PO_2_^−^)_phospholipids_	ν(CC)_glycogen_, ν(CO)
1092	ν_s_(PO_2_^−^)_B-DNA_			
1124	ν(CO)_RNA/ribose_	ν(CN)	ν(CC)_acyl (*trans* conformation)_	ν(CO), ν(CC)
1157		ν(CC), ν(CN), ρCH_2_)	ρ(CH_2_), ν(=C-C=)_conjugated_	ρ(CH_2_)
1172	(ν(CC)_ring_)_C,G,T_	δ(CH)_Tyr, Phe_		
1206		ν(CC)_Hyp, Phe, Tyr_		
1234		amide III/β-sheet		
1238	(ν_as_(PO_2_^−^))_B-DNA_			
1250		amide III/ random coil		
1252–1264	(ν(CC)_ring_)_A,T_	ω(CH_2_), δ(C=C-H)	ω(CH_2_),δ(C=C-H)_phospholipids_	ω(CH_2_), t(CH_2_)
1272		amide III/α-helix	ω(CH_2_), t(CH_2_)	ω(CH_2_), t(CH_2_)
1302	(ν(CC)_ring_)_RNA/A,C_			
1312	(ν(CC)_ring_)_G_	t(CH_2_)	t(CH_2_)	t(CH_2_)
1334	(ν(CC)_ring_)_G_			
1372 *^b^*	(ν(CC)_ring_)_A,G,T_	(δ_s_(CH_3_))_glycoproteins_	(δ_s_(CH_3_))_lipids/acyl chains_	(ω(CH_2_))_saccharides_
1400		t(NH_2_)		
1444–1450			α(CH_2_)	
1480 *^b^*		δ_s_(NH_3_^+^)		
1508		Tyr (ν(CC)_ring_)		
1546		((δ(CN-H)/ν(CN))_amide II_		
1552	(ν(CC)_ring_)_G_	(ν(CC)_ring_)_Trp_, ν(C=C)_porphyrin_		
1572–1574	(ν(CC)_ring_)_A,G_			
1580		ν(C=C), ν(C=N)	ν(C=C), ν(C=N)	
1602	(ν(CC)_ring_)_T_	(δ(C=CH))_Phe_		
1612–1690		amide I/β-sheet antiparallel		
1614	(ν(CC)_ring_)_A_, δ(NH_2_)_C_	ν(C=C)_Phe, Tyr, Trp_, α(NH_2_)		
1612–1690		amide I/β-sheet antiparallel		
1627–1640		amide I/β-sheet antiparallel		
1640–1650		amide I/random coil		
1650	δ(NH)_DNA_	ν(C=O)_amide I/__α__-helix_	ν(C=C)	
1690		ν(C=O)_amino acid side chain_		
1717	ν(C=O)_B-DNA_			
1730–42			(ν(C=O)_ester_)_phospholipids_	
2846–2854		ν_s_(CH_2_)	ν_s_(CH_2_)	ν_s_(CH_2_)
2873–2878		ν_s_(CH_3_)	ν_s_(CH_3_)	ν_s_(CH_3_)
2892		ν_as_(CH_2_)	ν_as_(CH_2_)	ν_as_(CH_2_)
2919		ν_as_(CH_2_)	ν_as_(CH_2_)	ν_as_(CH_2_)
2924–2930		ν_as_(CH_2_)	ν_as_(CH_2_)	ν_as_(CH_2_)
2954–2958		ν_as_(CH_3_)		
2964		ν_as_(CH_3_)	ν_as_(CH_3_)	ν_as_(CH_3_)
3070		amide B		
3250–3290		ν(NH)_amide A_		
3340–3370		ν(OH)		

The signals exclusively detected by either infrared or Raman are shaded in grey or in orange, respectively. ^*a*^ A—adenine; C—cytosine; dG—deoxyguanine; dT—deoxythymine; G—guanine; Hyp—hydroxyproline; Met—methionine; Phe—phenylalanine; Pro—proline; T—thymine; Trp—tryptophan; Tyr—tyrosine; U—uracil; Val—valine. δ—deformation; ϒ—out-of-plane deformation; τ—torsion; ν—stretching; α—scissoring; ρ—rocking; t—twisting; ω—wagging. s—symmetric; as—antisymmetric. (FTIR experimental fingerprint wavenumber range presented in this table were limited to 1000–1800 cm^−1^, whereas Raman experimental fingerprint wavenumber range is 600–1800 cm^−1^.), *^b^* Observed only for the MDA-MB-468 cell line.

**Table 2:** Confusion matrix of RF classification model on independent FTIR data (fingerprint region) using the 25% held-out validation method, and respective performance metrics (ROC area, sensitivity, specificity and overall accuracy) of the model towards each group of samples (true class).

		Classified as							
		MCF-7	MDA-MB-231	HCC-1143	MDA-MB-468	ROC AUC	Sensitivity (%)	Specificity (%)	Accuracy (%)
True class	MCF-7	415	0	11	22	0.97	92.6	90.9	91.4
	MDA-MB-231	0	450	1	0	0.99	99.7	100.0	99.9
	HCC-1143	31	0	347	15	0.97	88.3	97.6	95.2
	MDA-MB-468	71	0	17	192	0.96	68.6	97.1	92.0

**Table 3:** Confusion matrix of RF classification model on independent Raman data (fingerprint region) using the 25% held-out validation method, and respective performance metrics (ROC area, sensitivity, specificity and overall accuracy) of the model towards each group of samples (true class).

		Classified as							
		MCF-7	MDA-MB-231	HCC-1143	MDA-MB-468	ROC AUC	Sensitivity (%)	Specificity (%)	Accuracy (%)
True class	MCF-7	63	15	5	4	0.90	72.2	89.8	85.8
	MDA-MB-231	25	48	1	2	0.89	63.6	93.8	87.6
	HCC-1143	3	1	83	1	0.97	94.3	96.2	95.8
	MDA-MB-468	2	3	5	119	0.97	92.2	97.2	95.5

## Data Availability

The data presented in this study are available in article.

## Supplementary Materials

The following supporting information can be downloaded at: https://www.mdpi.com/article/10.3390/cancers14020452/s1. The Supplementary Materials contain the list of chemicals, as well as the details of the microRaman and microFTIR data acquisition, pre-processing and analysis. The Supplementary Materials also comprises Figure S1: Pre-processed and normalised FTIR (1000–1800 cm^−1^) and Raman (600–1800 cm^−1^) fingerprint spectra for each cell line. The average spectra are plotted in black. (a,b) MCF-7; (c,d) MDA-MB-231; (e,f) HCC-1143; (g,h) MDA-MB-468.

## References

[1] Turashvili G., Brogi E. (2017). Tumor Heterogeneity in Breast Cancer. Front. Med..

[2] Sung H., Ferlay J., Siegel R.L., Laversanne M., Soerjomataram I., Jemal A., Bray F. (2021). Global Cancer Statistics 2020: GLOBOCAN Estimates of Incidence and Mortality Worldwide for 36 Cancers in 185 Countries. CA Cancer J. Clin..

[3] Li C.I., Uribe D.J., Daling J.R. (2005). Clinical characteristics of different histologic types of breast cancer. Br. J. Cancer.

[4] Lazaro-Pacheco D., Shaaban A.M., Rehman S., Rehman I. (2019). Raman spectroscopy of breast cancer. Appl. Spectrosc. Rev..

[5] Polyak K. (2011). Heterogeneity in breast cancer. J. Clin. Investig..

[6] Bertos N.R., Park M. (2011). Breast cancer—One term, many entities?. J. Clin. Investig..

[7] Barzaman K., Karami J., Zarei Z., Hosseinzadeh A., Kazemi M.H., Moradi-Kalbolandi S., Safari E., Farahmand L. (2020). Breast cancer: Biology, biomarkers, and treatments. Int. Immunopharmacol..

[8] Luond F., Tiede S., Christofori G. (2021). Breast cancer as an example of tumour heterogeneity and tumour cell plasticity during malignant progression. Br. J. Cancer.

[9] Liu M.C., Pitcher B.N., Mardis E.R., Davies S.R., Friedman P.N., Snider J.E., Vickery T.L., Reed J.P., DeSchryver K., Singh B. (2016). PAM50 gene signatures and breast cancer prognosis with adjuvant anthracycline- and taxane-based chemotherapy: Correlative analysis of C9741 (Alliance). NPJ Breast Cancer.

[10] Lehmann B.D., Bauer J.A., Chen X., Sanders M.E., Chakravarthy A.B., Shyr Y., Pietenpol J.A. (2011). Identification of human triple-negative breast cancer subtypes and preclinical models for selection of targeted therapies. J. Clin. Investig..

[11] Lehmann B.D., Jovanovic B., Chen X., Estrada M.V., Johnson K.N., Shyr Y., Moses H.L., Sanders M.E., Pietenpol J.A. (2016). Refinement of Triple-Negative Breast Cancer Molecular Subtypes: Implications for Neoadjuvant Chemotherapy Selection. PLoS ONE.

[12] Dai X., Cheng H., Bai Z., Li J. (2017). Breast Cancer Cell Line Classification and Its Relevance with Breast Tumor Subtyping. J. Cancer.

[13] Hubalek M., Czech T., Muller H. (2017). Biological Subtypes of Triple-Negative Breast Cancer. Breast Care.

[14] Fougner C., Bergholtz H., Norum J.H., Sorlie T. (2020). Re-definition of claudin-low as a breast cancer phenotype. Nat. Commun..

[15] Kassam F., Enright K., Dent R., Dranitsaris G., Myers J., Flynn C., Fralick M., Kumar R., Clemons M. (2009). Survival outcomes for patients with metastatic triple-negative breast cancer: Implications for clinical practice and trial design. Clin. Breast Cancer.

[16] Garrido-Castro A.C., Lin N.U., Polyak K. (2019). Insights into Molecular Classifications of Triple-Negative Breast Cancer: Improving Patient Selection for Treatment. Cancer Discov..

[17] Foulkes W.D., Smith I.E., Reis-Filho J.S. (2010). Triple-Negative Breast Cancer. N. Engl. J. Med..

[18] Yin L., Duan J.J., Bian X.W., Yu S.C. (2020). Triple-negative breast cancer molecular subtyping and treatment progress. Breast Cancer Res..

[19] Dent R., Trudeau M., Pritchard K.I., Hanna W.M., Kahn H.K., Sawka C.A., Lickley L.A., Rawlinson E., Sun P., Narod S.A. (2007). Triple-negative breast cancer: Clinical features and patterns of recurrence. Clin. Cancer Res..

[20] Bento M.J., Goncalves G., Aguiar A., Castro C., Veloso V., Rodrigues V. (2015). Performance indicators evaluation of the population-based breast cancer screening programme in Northern Portugal using the European Guidelines. Cancer Epidemiol..

[21] Zolota V., Tzelepi V., Piperigkou Z., Kourea H., Papakonstantinou E., Argentou M.-I., Karamanos N.K. (2021). Epigenetic Alterations in Triple-Negative Breast Cancer-The Critical Role of Extracellular Matrix. Cancers.

[22] Plodinec M., Loparic M., Monnier C.A., Obermann E.C., Zanetti-Dallenbach R., Oertle P., Hyotyla J.T., Aebi U., Bentires-Alj M., Lim R.Y. (2012). The nanomechanical signature of breast cancer. Nat. Nanotechnol..

[23] Xu W., Mezencev R., Kim B., Wang L., McDonald J., Sulchek T. (2012). Cell stiffness is a biomarker of the metastatic potential of ovarian cancer cells. PLoS ONE.

[24] Lekka M. (2016). Discrimination between Normal and Cancerous Cells Using AFM. Bionanoscience.

[25] Marques M.P.M., Batista de Carvalho A.L.M., Mamede A.P., Dopplapudi A., Rudic S., Tyagi M., Garcia Sakai V., Batista de Carvalho L.A.E. (2020). A New Look into the Mode of Action of Metal-Based Anticancer Drugs. Molecules.

[26] Baker M.J., Trevisan J., Bassan P., Bhargava R., Butler H.J., Dorling K.M., Fielden P.R., Fogarty S.W., Fullwood N.J., Heys K.A. (2014). Using Fourier transform IR spectroscopy to analyze biological materials. Nat. Protoc..

[27] Grosserueschkamp F., Kallenbach-Thieltges A., Behrens T., Brüning T., Altmayer M., Stamatis G., Theegarten D., Gerwert K. (2015). Marker-free automated histopathological annotation of lung tumour subtypes by FTIR imaging. Analyst.

[28] Haka A.S., Shafer-Peltier K.E., Fitzmaurice M., Crowe J., Dasari R.R., Feld M.S. (2005). Diagnosing breast cancer by using Raman spectroscopy. Proc. Natl. Acad. Sci. USA.

[29] Medeiros P.S., Batista de Carvalho A.L.M., Ruano C., Otero J.C., Marques M.P. (2016). Raman microspectroscopy for probing the impact of a dietary antioxidant on human breast cancer cells. Food Funct..

[30] Butler H.J., Ashton L., Bird B., Cinque G., Curtis K., Dorney J., White K.E., Fullwood N.J., Gardner B., Martin-Hirsch P.L. (2016). Using Raman spectroscopy to characterise biological materials. Nat. Protoc..

[31] Hughes C., Baker M.J. (2016). Can mid-infrared biomedical spectroscopy of cells, fluids and tissue aid improvements in cancer survival? A patient paradigm. Analyst.

[32] Pilling M., Gardner P. (2016). Fundamental developments in infrared spectroscopic imaging for biomedical applications. Chem. Soc. Rev..

[33] Batista de Carvalho A.L.M., Pilling M., Gardner P., Doherty J., Cinque G., Wehbe K., Kelley C., Batista de Carvalho L.A.E., Marques M.P. (2016). Chemotherapeutic response to cisplatin-like drugs in human breast cancer cells probed by vibrational microspectroscopy. Faraday Discuss..

[34] Santos I.P., Barroso E.M., Bakker Schut T.C., Caspers P.J., van Lanschot C.G.F., Choi D.-H., van der Kamp M.F., Smits R.W.H., van Doorn R., Verdijk R.M. (2017). Raman spectroscopy for cancer detection and cancer surgery guidance: Translation to the clinics. Analyst.

[35] Mohamed H.T., Untereiner V., Proult I., Ibrahim S.A., Götte M., El-Shinawi M., Mohamed M.M., Sockalingum G.D., Brezillon S. (2018). Characterization of inflammatory breast cancer: A vibrational microspectroscopy and imaging approach at the cellular and tissue level. Analyst.

[36] Schie I.W., Rüger J., Mondol A.S., Ramoji A., Neugebauer U., Krafft C., Popp J. (2018). High-Throughput Screening Raman Spectroscopy Platform for Label-Free Cellomics. Anal. Chem..

[37] Marques M.P.M., Batista de Carvalho A.L.M., Mamede A.P., Santos I.P., Garcia Sakai V., Dopplapudi A., Cinque G., Wolna M., Gardner P., Batista de Carvalho L.A.E. (2019). Chemotherapeutic Targets in Osteosarcoma: Insights from Synchrotron-MicroFTIR and Quasi-Elastic Neutron Scattering. J. Phys. Chem. B.

[38] Elumalai S., Manago S., De Luca A.C. (2020). Raman Microscopy: Progress in Research on Cancer Cell Sensing. Sensors.

[39] Quaroni L. (2020). Infrared microscopy in the study of cellular biochemistry. Infrared Phys. Technol..

[40] Mohamed H.T., Untereiner V., Cinque G., Ibrahim S.A., Götte M., Nguyen N.Q., Rivet R., Sockalingum G.D., Brezillon S. (2020). Infrared Microspectroscopy and Imaging Analysis of Inflammatory and Non-Inflammatory Breast Cancer Cells and Their GAG Secretome. Molecules.

[41] Su K.Y., Lee W.L. (2020). Fourier Transform Infrared Spectroscopy as a Cancer Screening and Diagnostic Tool: A Review and Prospects. Cancers.

[42] Gardner B., Matousek P., Stone N. (2021). Self-absorption corrected non-invasive transmission Raman spectroscopy (of biological tissue). Analyst.

[43] Zhang L., Li C., Peng D., Yi X., He S., Liu F., Zheng X., Huang W.E., Zhao L., Huang X. (2022). Raman spectroscopy and machine learning for the classification of breast cancers. Spectrochim. Acta A Mol. Biomol. Spectrosc..

[44] Iwasaki K., Araki A., Krishna C.M., Maruyama R., Yamamoto T., Noothalapati H. (2021). Identification of Molecular Basis for Objective Discrimination of Breast Cancer Cells (MCF-7) from Normal Human Mammary Epithelial Cells by Raman Microspectroscopy and Multivariate Curve Resolution Analysis. Int. J. Mol. Sci..

[45] Zoladek A., Pascut F.C., Patel P., Notingher I. (2011). Non-invasive time-course imaging of apoptotic cells by confocal Raman micro-spectroscopy. J. Raman Spectrosc..

[46] Wehbe K., Filik J., Frogley M.D., Cinque G. (2013). The effect of optical substrates on micro-FTIR analysis of single mammalian cells. Anal. Bioanal. Chem..

[47] Notingher I., Verrier S., Haque S., Polak J.M., Hench L.L. (2002). Spectroscopic Study of Human Lung Epithelial Cells (A549) in Culture: Living Cells Versus Dead Cells. Biopolymers.

[48] Talari A.C.S., Evans C.A., Holen I., Coleman R.E., Rehman I.U. (2015). Raman spectroscopic analysis differentiates between breast cancer cell lines. J. Raman Spectrosc..

[49] Marro M., Nieva C., Sanz-Pamplona R., Sierra A. (2014). Molecular monitoring of epithelial-to-mesenchymal transition in breast cancer cells by means of Raman spectroscopy. Biochim. Biophys. Acta.

[50] Ozek N.S., Tuna S., Erson-Bensan A.E., Severcan F. (2010). Characterization of microRNA-125b expression in MCF7 breast cancer cells by ATR-FTIR spectroscopy. Analyst.

[51] Dolce V., Rita Cappello A., Lappano R., Maggiolini M. (2011). Glycerophospholipid synthesis as a novel drug target against cancer. Curr. Mol. Pharmacol..

[52] Chaturvedi D., Balaji S.A., Bn V.K., Ariese F., Umapathy S., Rangarajan A. (2016). Different Phases of Breast Cancer Cells: Raman Study of Immortalized, Transformed, and Invasive Cells. Biosensors.

[53] Abramczyk H., Surmacki J., Kopec M., Olejnik A.K., Kaufman-Szymczyk A., Fabianowska-Majewska K. (2016). Epigenetic changes in cancer by Raman imaging, fluorescence imaging, AFM and scanning near-field optical microscopy (SNOM). Acetylation in normal and human cancer breast cells MCF10A, MCF7 and MDA-MB-231. Analyst.

[54] Ross D.T., Scherf U., Eisen M.B., Perou C.M., Rees C., Spellman P., Iyer V., Jeffrey S.S., Van de Rijn M., Waltham M. (2000). Systematic variation in gene expression patterns in human cancer cell lines. Nat. Genet..

[55] Sellappan S., Grijalva R., Zhou X., Yang W., Bar Eli M., Mills G.B., Yu D. (2004). Lineage Infidelity of MDA-MB-435 Cells: Expression of Melanocyte Proteins in a Breast Cancer Cell Line. Cancer Res..

[56] Rae J.M., Ramus S.J., Waltham M., Armes J.E., Campbell I.G., Clarke R., Barndt R.J., Johnson M.D., Thompson E.W. (2004). Common origins of MDA-MB-435 cells from various sources with those shown to have melanoma properties. Clin. Exp. Metastasis.

[57] Rae J.M., Creighton C.J., Meck J.M., Haddad B.R., Johnson M.D. (2007). MDA-MB-435 cells are derived from M14 melanoma cells--a loss for breast cancer, but a boon for melanoma research. Breast Cancer Res. Treat..

[58] Kenny P.A., Lee G.Y., Myers C.A., Neve R.M., Semeiks J.R., Spellman P.T., Lorenz K., Lee E.H., Barcellos-Hoff M.H., Petersen O.W. (2007). The morphologies of breast cancer cell lines in three-dimensional assays correlate with their profiles of gene expression. Mol. Oncol..

[59] Ziperstein M.J., Guzman A., Kaufman L.J. (2015). Breast Cancer Cell Line Aggregate Morphology Does Not Predict Invasive Capacity. PLoS ONE.

[60] Koren S., Bentires-Alj M. (2015). Breast Tumor Heterogeneity: Source of Fitness, Hurdle for Therapy. Mol. Cell.

[61] Prat A., Adamo B., Cheang M.C., Anders C.K., Carey L.A., Perou C.M. (2013). Molecular characterization of basal-like and non-basal-like triple-negative breast cancer. Oncologist.

[62] Zhang X., Chan T., Mak M. (2021). Morphodynamic signatures of MDA-MB-231 single cells and cell doublets undergoing invasion in confined microenvironments. Sci. Rep..

