# Supplementary material for: Who’s Who? Discrimination of Human Breast Cancer Cell Lines by Raman and FTIR Microspectroscopy
Source: Cancers (Basel). 2022 Jan 17;14(2):452. doi: 10.3390/cancers14020452 (PMC8773714; doi:10.3390/cancers14020452)
Supplement: Supplementary file 1 [file cancers-14-00452-s001.zip › cancers-1511211-supplementary.pdf]

Who's Who? Discrimination of Human Breast Cancer Cell Lines by Raman and FTIR Microspectroscopy

I.P. Santos, C.B. Martins, L.A.E. Batista de Carvalho, M.P.M. Marques and A.L.M. Batista de Carvalho

TABLE OF CONTENTS

1. Materials and Methods
   1.1. Chemicals
   1.2. Cell Culture
   1.3. Raman Microspectroscopy
   1.4. FTIR Microspectroscopy
   1.5. Pre-processing of Infrared and Raman Spectra
      1.5.1. Infrared data pre-processing
      1.5.2. Raman data pre-processing
   1.6. Data Analysis
      1.6.1. Hierarchical clustering analysis
      1.6.2. Principal component analysis
      1.6.3. PCA-Random Forest model and validation

2. Figures

   Figure S1 – Pre-processed and normalized FTIR and Raman spectra of each cell line.

3. References

1. Materials and Methods

1.1. Chemicals

Antibiotics (penicillin–streptomycin 100x solution), Dulbecco's Modified Eagle's Medium-high glucose (DMEM-HG, 4500 mgL^{-1} glucose), ethylenediaminetetraacetic acid (EDTA, disodium salt, dihydrate), formalin (10% *v/v* neutral-buffered formalin, *ca.* 4% formaldehyde), phosphate buffered saline (PBS), trypan blue (0.4%), trypsin (10x solution, 25 g porcine trypsin per liter in 0.9% sodium chloride), and inorganic salts and acids (of analytical grade) were purchased from Sigma-Aldrich Chemical S.A. (Sintra, Portugal). Fetal bovine serum (FBS) was obtained from Gibco-Life Technologies (Porto, Portugal).

1.2. Cell Culture

Four human breast cancer cells were investigated in this work: (i) triple negative (TNBC) – MDA-MB-231 ductal carcinoma, claudin-low/MSL, metastatic; MDA-MB-468 ductal carcinoma, BL1, metastatic; and HCC-1143 invasive ductal carcinoma, non-metastatic, BL1; (ii) non-triple-negative – MCF-7 adenocarcinoma, luminal A/HER2 negative, metastatic.

MDA-MB-231, MCF-7 and HCC-1143 cell lines where purchased from the Leibniz Institute DSMZ-German Collection of Microorganisms and Cell Cultures, while MDA-MB-468 cell line was provided by Dr João Nuno Moreira from the Centre for Neurosciences and Cellular Biology & Centre for Innovative Biomedicine and Biotechnology of the University of Coimbra.

The cells were cultured as monolayers at 37 °C, in a humidified atmosphere of 5% CO_2. The cultures were maintained in RPMI-1640 medium supplemented with 10% *(v/v)* heat-inactivated FBS, 1% *(v/v)* penicillin (100 U/mL)/streptomycin (100 mg/mL) and sodium bicarbonate-20 mM (pH 7.4). Cells were subcultured at 80% confluence, using trypsin-EDTA (1x). Under these conditions, the duplication time was 26 h. The cells were always in the logarithmic phase of growth when the tested agents were added.

1.3. Raman Microspectroscopy

The Raman spectra were recorded in a WITec confocal Raman microscope system alpha 300R (WITec GmbH, Germany) with an automated sample stage, coupled to an ultrahigh-throughput spectrometer UHTS 300Vis-NIR (f/4, 300mm focal length and a 600 lines/mm grating) chosen in order to record the complete Raman spectra, fingerprint and high wavenumber spectral regions.

The excitation radiation was a 532 nm line of a frequency doubled Nd:YAG laser, with a maximum output power of 15 mW at the sample position. The laser light was focused on the sample using an 100X Zeiss EC "Epiplan-Neofluar" objective (NA 0.80, WD 1.3 mm). The detector was a 1650x200 pixels thermoelectrically cooled CCD Camera ANDOR iDUS DV401A-BV-352, peltier-cooled down to -60°C, front-illuminated chip with NIR/VIS anti-reflection coating and spectral resolution <0.8 cm^{-1}/pixel. Each spectrum was obtained with 3 accumulations and 15 s of exposure time.

1.4. FTIR Microspectroscopy

The microFTIR spectra were acquired in the mid-IR interval 400-4000 cm^{-1}, in transmission mode, using a Bruker Optics Vertex 70 FTIR spectrometer purged by CO_2-free dry air, coupled to a Hyperion 3000 microscope with a 15x objective (NA=0.4). A KBr beamsplitter and a LN_2-cooled wide-band mercury cadmium telluride (MCT) detector (50x50 μm^2 area) were used.Each spectrum corresponded to the average of two measurements of 32 scans each, at a 4 cm^{-1} resolution. The error in wavenumbers was estimated to be less than 1 cm^{-1}. Background was acquired every 10 spectra. All samples were prepared in triplicate, in a single experiment.

1.5. Pre-processing of Infrared and Raman Spectra

1.5.1. Infrared data pre-processing

Infrared spectra were quality checked (to remove spectra with high levels of scattering and fixed-pattern noise) based on the amide I band intensity, spectra with absorbance between 0.1 and 1 being retained. The data was corrected using the resonant Mie scattering with EMSC algorithm (20 iterations), cropped to the 1000−3800 cm^{-1} wavenumber region and vector normalised [1]. This correction was performed independently for each separated spectrum, since these may have experienced a different distortion due to the heterogeneous nature of the samples at distinct locations from which data was collected. Initial pre-processing was based on a PC-based noise reduction algorithm (with 40 PC´s), achieved by retaining the selected number of principal components and then recombing the dataset. FTIR spectra were cropped into two spectral ranges: fingerprint region (1000-1800 cm^{-1}) and high-wavenumber interval (2700-3650 cm^{-1}). The spectra were vector normalised and mean-centred before PCA analysis (Figure S1).

1.5.2. Raman data pre-processing

The Raman spectra were initially cropped to the pre-processing spectral range 400−3700 cm^{-1}, and the signal background generated by optical elements was subtracted. The data were quality checked based on the fingerprint integrated signal (1000−1490 cm^{-1}) and on the CH band integrated signal (2800−3050 cm^{-1}) spectra, with a signal-to-noise ratio below 10 being retained. Identification and removal of cosmic ray events was performed using a first-order derivative. A principal-component (PC)-based noise reduction algorithm was applied by retaining a selected number of principal components (40 PC's) and then recombing the dataset.

All spectra were then cropped into two spectral regions: fingerprint region (600−1800 cm^{-1}) and high-wavenumber region (2700−3150 cm^{-1}). Scaling to the average of all individual spectra was performed, using the Extended Multiplicative Signal Correction (EMSC) algorithm [2] with a 0th order polynomial background (Figure S1).

1.6. Data Analysis

1.6.1. Hierarchical clustering analysis

An unsupervised hierarchical clustering-derived dendrogram of the mean fingerprint FTIR and Raman spectra was performed. The Euclidean distance between the average spectra was initially computed, for each cell line, from which an agglomerative hierarchical cluster tree was generated.

1.6.2. Principal component analysis

All Raman and FTIR spectra were vector normalised and mean-centred before analysis. An unsupervised method of multivariate analysis – principal component analysis (PCA) – was carried out on the individual spectra (and separately for each spectral region probed) with standard singular value decomposition of the data, using MATLAB 2020b (The MathWorks Inc., MA, USA). The order of the principal components (PCs) denotes their importance to the dataset, PC-1 corresponding to the highest amount of variation.

1.6.3. PCA-Random Forest model and validation

A Random Forest (RF) classification model was applied (using MATLAB 2020b) to assess the discriminatory power of infrared and Raman spectral information, with a view to distinguish between: (1) all studied cell lines; (2) triple-negative breast cancer (TNBC) *vs* non-TNBC; (3) TNBC cell-lines (mesenchymal stem-like *vs* basal-like 1) and (4) TNBC basal-like 1 cell lines with high-metastatic potential *vs* low-metastatic potential. For each RF classification model, data was randomly divided into two groups: 75% of the spectra to train the model and 25% for independently validation. Prior to model creation, PCA was performed to reduce the data dimensionality, the scores of the first four PCs having been used as input to create the RF models. These were generated with 100 tree learners each one with 10 maximum branch points.

For each classification task a confusion table was generated, displaying the number of predicted spectra *vs* true classes. Specificity was defined as the fraction of correctly predicted negatives from the total number of true negatives. Sensitivity was calculated as the fraction of correctly predicted positives from the total number of true positives. The area under the receiver-characteristic operator curve (ROC) measures the performance of the classification model. Overall accuracy was calculated as the fraction of correctly predicted spectra (true positives and true negatives) from the total number of spectra tested.

Validation of FTIR classification model was performed using an independent test set, comprising 25% of the spectra from each class, in a total of 1572 spectra: 448 spectra from MCF-7, 451 spectra from MDA-MB-231, 393 spectra from HCC-1143, and 280 spectra from MDA-MB-468.

Validation of Raman classification model was performed using an independent test set, comprising 25% of the spectra from each class, in a total of 380 spectra: 87 spectra from MCF-7, 76 spectra from MDA-MB-231, 88 spectra from HCC-1143, and 129 spectra from MDA-MB-468.

2. Figures

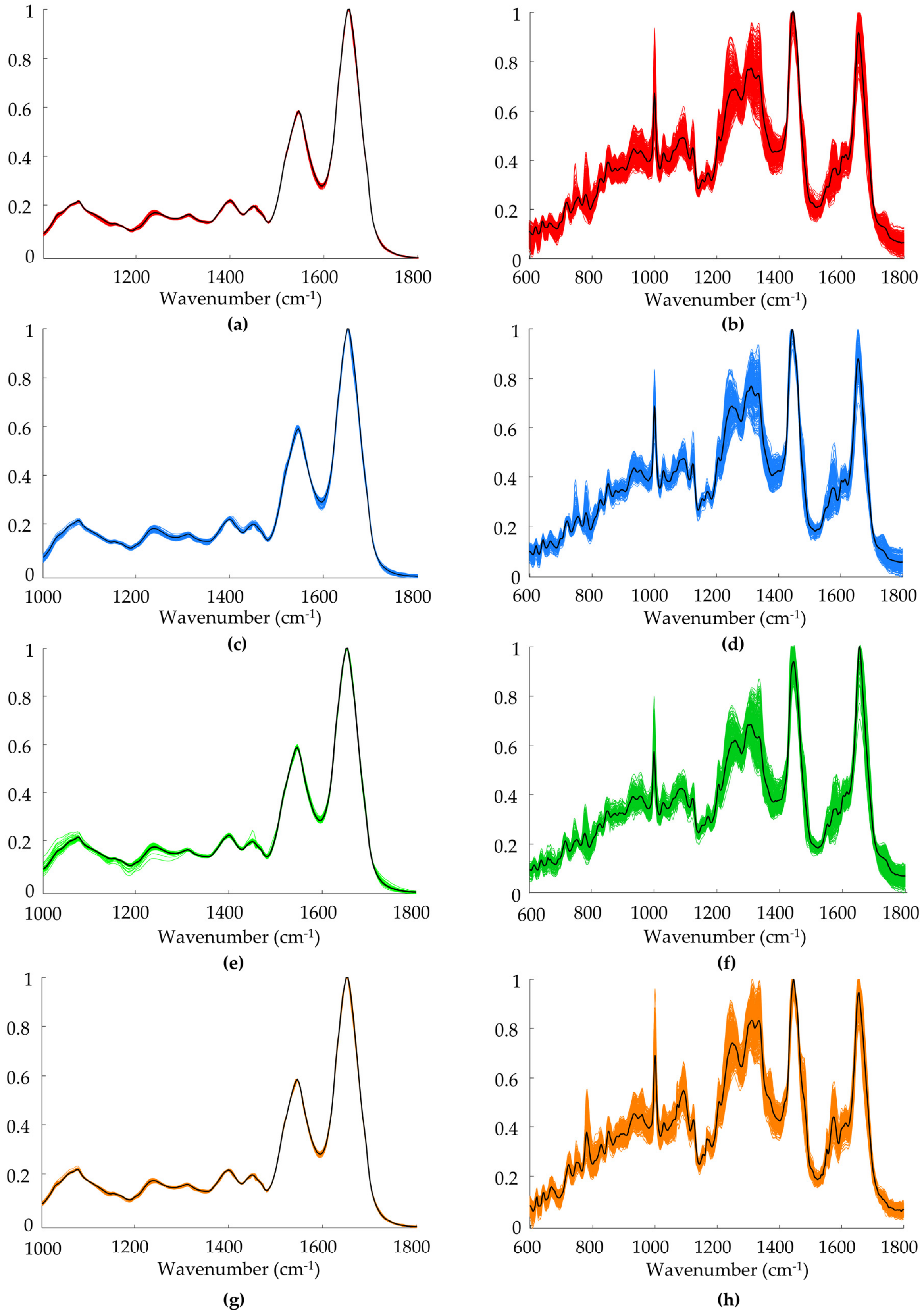

Figure S1. Pre-processed and normalized fingerprint FTIR (1000-1800 cm^{-1}) and Raman (600-1800 cm^{-1}) spectra of each cell line. The average spectrum is plotted in black. **(a-b)** MCF-7; **(c-d)** MDA-MB-231; **(e-f)** HCC-1143; **(g-h)** MDA-MB-468.

3. References

[1] P. Bassan *et al.*, "FTIR microscopy of biological cells and tissue: data analysis using resonant Mie scattering (RMieS) EMSC algorithm," *Analyst,* vol. 137, no. 6, pp. 1370-7, Mar 21 2012, doi: 10.1039/c2an16088a.

[2] H. Martens and E. Stark, "Extended multiplicative signal correction and spectral interference subtraction: new preprocessing methods for near infrared spectroscopy," (in eng), *J Pharm Biomed Anal,* vol. 9, no. 8, pp. 625-35, 1991, doi: 10.1016/0731-7085(91)80188-f.
